# Life-Long Physical Activity Involvement and the Risk of Ischemic Stroke in Southern China

**DOI:** 10.4061/2010/415241

**Published:** 2011-01-17

**Authors:** Andy H. Lee, Wenbin Liang

**Affiliations:** ^1^School of Public Health, Curtin Health Innovation Research Institute, Curtin University, GPO Box U 1987, Perth, WA 6845, Australia; ^2^National Drug Research Institute, Curtin University, GPO Box U 1987, Perth, WA 6845, Australia

## Abstract

A case-control study was conducted in southern China to investigate the relationship between life-long physical activity involvement and the risk of ischemic stroke. Information on life-long physical activity exposure and other lifestyle characteristics was obtained from 374 incident stroke patients and 464 hospital-based controls using a validated and reliable questionnaire. Logistic regression analyses were performed to assess the association between life-long physical activity involvement and the ischemic stroke risk. The control subjects reported more involvement in physical activity over the life course than the stroke patients (*P* < .001). The risk of ischemic stroke was inversely associated with life-long physical activity exposure, with adjusted odds ratio 0.39 (95% confidence interval 0.25 to 0.59) for participants who had always been involved relative to those who have never been much involved. The dose-response relationship was also significant (*P* < .001). Therefore, being active life long should be encouraged to prevent this major chronic disease.

## 1. Introduction

Stroke is a major cause of illness, death, and health expenditures. Ischemic stroke accounts for more than 70% of all stroke cases in western countries, and about 60% in China [1, 2]. Because of the high burden and societal cost associated with stroke, the need for effective primary preventive strategy is imperative [3].

The health benefits of physical activity are well documented. Physical activity is a modifiable lifestyle factor for many chronic diseases including cardiovascular diseases, diabetes mellitus, osteoporosis, and some cancers [4]. It is estimated that half of all functional decline associated with the ageing process is preventable if adequate levels of physical activity are maintained [5], confirming the contribution of physical activity to increasing longevity and improving survival [6]. 

Despite some conflicting results, the majority of scientific evidences have demonstrated the value of physical activity for stroke prevention [7, 8]. In particular, large-scale prospective cohort studies [9–11] as well as meta-analyses [12–14] have concluded that physical activity is associated with reduced risk of ischemic stroke in a dose-response manner. Moreover, for patients with first-ever stroke, high levels of recent physical activity can lessen the disease severity and lead to a better long-term outcome [15, 16]. 

Unlike previous studies that focused on occupational and/or leisure-time activities during adulthood, the present study aimed to ascertain whether life-long physical activity involvement is associated with a reduced risk of ischemic stroke, which has not been reported in the literature. It formed part of a research project assessing the role of dietary and lifestyle factors for the prevention of this major disease. 

## 2. Materials and Methods

### 2.1. Study Design and Subjects

A hospital-based case-control study was conducted in the Guangdong Province of Southern China between July 2007 and July 2008. Subjects were recruited from three teaching hospitals in Foshan city, namely, the First People's Hospital of Shunde, First People's Hospital of Nanhai, and Second People's Hospital of Foshan. Cases were incident ischemic stroke patients referred from the inpatient wards of the neurology departments. Controls were recruited from outpatient clinics of the departments of gastroenterology, dermatology, Chinese medicine, urology, and otolaryngology. To be eligible, subjects must have resided in Foshan for at least the past five years and were alive at the time of their interview. Inclusion criteria for ischemic stroke cases were sudden onset of a focal neurological event, with symptoms lasting for more than 24 hours and subsequent confirmation of infarction in the brain by CT or MRI scans, and no previous history of stroke. Therefore, only patients with a first-ever ischemic stroke (thrombotic or embolic) were considered. Fatal cases due to stroke were excluded because of ethical constraints. An eligible control had neither a history nor clinical evidence indicating a previous stroke, and whose treatment at the outpatient department was not related to cardiovascular disease, a malignant tumour, or diabetes. Controls were frequency matched to cases within 5 years of age and recruited during the same period as cases. A total of 500 incident ischemic stroke patients and 600 eligible controls were initially approached.

### 2.2. Interview

The hospital neurology wards notified the second author within two days of each stroke patient admission. A face-to-face interview was then arranged soon after and before discharge from hospital. Control subjects were interviewed at the outpatient clinics. All consented participants were assured of confidentiality and their right to withdraw without prejudice. Whenever a case was unable to answer because of the morbidity caused by stroke, responses were sought from his or her next-of-kin instead. The validity and reliability of using such proxy information has been established [17]. The project protocol was approved by the three participating hospitals and the Human Research Ethics Committee of Curtin University.

### 2.3. Exposure Measurements

A structured questionnaire was administered to obtain demographic and lifestyle characteristics including age, gender, weight (kg), height (m), education level (primary school; secondary school or above), smoking status (nonsmoker; current/former smoker) and pack-years, and alcohol drinking status (nondrinker; drinker). Self-reported height and weight measurements and previous history of hypertension, hyperlipidemia, and diabetes, were confirmed with medical records whenever available. Information on life-long physical activity involvement was sought, defined as “doing active sports or vigorous exercise long enough to get sweaty, at least twice a week”, over the entire life course [18]. Response options were: “never been much involved”, “previously active, but not any more”, “active just recently”, “intermittently active”, “always been involved” [18].

### 2.4. Statistical Analysis

Characteristics of participants were summarized using descriptive statistics. Unconditional logistic regression analyses were then performed to estimate the effects of life-long physical activity involvement on the ischemic stroke risk. In addition to reporting crude and adjusted odds ratios (ORs) and associated 95% confidence intervals (CIs), tests for linear trend were conducted to assess the dose-response relationship for both genders. Other independent variables included in the multivariable models were age, education level, body mass index (BMI = weight/height^2^), smoking status, smoking pack-years, alcohol drinking status, presence of hypertension, hyperlipidemia, and diabetes. These variables were either plausible risk factors from the literature or considered potential confounders based on our univariate analysis. Statistical analyses were undertaken using the STATA package release 10 (Stata Corporation, Texas, USA).

## 3. Results

A total of 427 cases and 512 controls satisfying the selection criteria signed the consent form and agreed to participate. Among them, 374 (226 male, 148 female) patients and 464 (248 male, 216 female) control subjects completed the interview, representing a final response rate of 74.8% and 77.3%, respectively. No significant differences in age and gender distributions were found between participants and nonparticipants. Proxy information was used in 22% of the stroke cases, including patients with aphasia.

Characteristics of subjects by gender and case-control status are shown in Table 1. The mean age and BMI were similar between cases and controls. The stroke patients had a higher prevalence of hypertension and diabetes for both genders. They were more likely to be smokers, and had a higher cumulative smoking exposure than their counterparts without the disease. In relation to life-long physical activity, the two groups were significantly different for both genders (*P* < .001) and separately for males (*P* < .001) and females (*P* = .025). The prevalence estimates suggested that the control subjects were more active over the life course than the stroke patients. 

Table 2 presents the results of the multivariable logistic regression analyses. The risk of ischemic stroke was inversely associated with life-long physical activity exposure, with adjusted OR 0.39 (95% CI 0.25 to 0.59) for participants who had always been involved relative to those never been much involved, after accounting for the effects of confounding factors. The corresponding dose-response relationship was also significant (*P* < .001) according to the linear trend test. Separate analyses by gender confirmed the inverse association, but the results appeared to be more significant among males than females.

## 4. Discussion

This was the first study to investigate the impact of life-long physical activity involvement on the ischemic stroke risk. The sample size of 838 subjects (373 cases, 464 controls) provided sufficient power for the multivariable analysis. To ensure correct classification of the disease status, we recruited only incident first-ever ischemic stroke patients with brain image confirmed, and all controls were screened for possible stroke symptoms by a neurologist. 

This study showed that the Chinese stroke patients were less active over the life course than people without the disease. The finding of significant inverse association between long-term physical activity exposure and risk of ischemic stroke, independent of other risk factors, was consistent with previous epidemiologic studies [9–11] and meta-analyses [12–14] which documented the risk reduction due to occupational and leisure-time physical activities during adulthood. The protective effect of regular physical activity is biologically plausible. Experimental studies have shown that physical activity can upregulate endothelial nitric oxide synthase in the vasculature [19] and improve endothelial function [20]. 

Several limitations should be considered when interpreting the findings. The assessment of life-long physical activity exposure was based on self-report, unlike physical activity in daily life which can be objectively measured using instruments such as pedometers and triaxial accelerometers. Consequently, the subjective responses received might incur some perception bias by the participants. Face-to-face interviews were thus used to help interpretation and to improve the accuracy of their answers. Moreover, the same investigator (second author) conducted all interviews to eliminate interinterviewer bias. The control subjects were recruited during the same period and from the same catchment area as the cases and should be representative of the southern Chinese population, but the inherent selection bias could not be avoided because of their voluntary participation in the study. The potential protective effect of life-long physical activity was not established for ischemic stroke and all participants were blinded to the study hypothesis so that information bias was unlikely. Nevertheless, residual confounding might still present even though plausible confounding factors were controlled for in the multivariable analyses.

## 5. Conclusion

Further replications of the study in other populations and other stroke subtypes are recommended. In the mean time, physical activity should be encouraged and maintained throughout the life course because of its potential benefit in preventing this major chronic disease.

## Figures and Tables

**Table 1:** Characteristic of subjects by gender and case-control status.

Variable	Male (*n* = 474)		Female (*n* = 364)	
	Case (*n* = 226)	Control (*n* = 248)	Case (*n* = 148)	Control (*n* = 216)
Mean age (SD) years	69.6 (8.0)	68.7 (7.0)	69.1 (9.2)	69.0 (9.0)
Mean BMI (SD) kg/m^2^	22.9 (2.7)	23.1 (3.0)	21.5 (3.6)	22.8 (3.6)
Primary school education: *n* (%)	104 (46%)	88 (35.5%)	82 (55.4%)	103 (47.7%)
Hypertension: *n* (%)	119 (52.7%)	71 (28.6%)	76 (51.4%)	60 (27.8%)
Hyperlipidemia: *n* (%)	51 (22.6%)	23 (9.3%)	17 (11.5%)	36 (16.7%)
Diabetes: *n* (%)	48 (21.2%)	8 (3.2%)	31 (21%)	4 (1.9%)
Alcohol drinker: *n* (%)	157 (69.5%)	147 (59.3%)	13 (8.8%)	43 (19.9%)
Smoker: *n* (%)	162 (71.7%)	139 (56.1%)	15 (10.1%)	11 (5.1%)
Mean smoking (SD) pack-years	18.5 (22.1)	17.1 (22.2)	1.3 (6.8)	1.0 (5.5)

Life-long physical activity: *n* (%)				
never been much involved	71 (31.4%)	56 (22.6%)	60 (40.5%)	63 (29.2%)
previously but not any more	84 (37.2%)	53 (21.4%)	39 (26.4%)	49 (22.7%)
active just recently	12 (5.3%)	15 (6.1%)	10 (6.8%)	19 (8.8%)
intermittently active	22 (9.7%)	29 (11.7%)	6 (4.1%)	25 (11.6%)
always been involved	37 (16.4%)	95 (38.3%)	33 (22.3%)	60 (27.8%)

BMI: body mass index.

**Table 2:** Life-long physical activity and risk of ischemic stroke in southern China.

Life-long physical activity	Crude OR	95% CI		*P*	Adjusted OR*	95% CI		*P*
Both genders						*P* for trend <.001		
Never been much involved	1				1			
Previously but not any more	1.10	(.76, 1.57)		.62	1.10	(.73, 1.64)		.65
Active just recently	0.59	(.33, 1.06)		.08	0.52	(.26, 1.02)		.06
Intermittently active	0.47	(.28, .79)		.01	0.40	(.22, .73)		.01
Always been involved	0.41	(.28, .60)		<.01	0.39	(.25, .59)		<.01

Male						*P* for trend <.001		
Never been much involved	1				1			
Previously but not any more	1.25	(.77, 2.04)		.37	1.26	(.73, 2.17)		.41
Active just recently	0.63	(.27, 1.46)		.28	0.57	(.22, 1.50)		.26
Intermittently active	0.60	(.31, 1.15)		.13	0.46	(.21, .99)		.05
Always been involved	0.31	(.18, .51)		<.01	0.29	(.16, .51)		<.01

Female						*P* for trend = .017		
Never been much involved	1				1			
Previously but not any more	0.84	(.48, 1.45)		.52	0.95	(.51, 1.78)		.87
Active just recently	0.55	(.24, 1.28)		.17	0.55	(.21, 1.46)		.23
Intermittently active	0.25	(.10, .66)		.01	0.25	(.08, .75)		.01
Always been involved	0.58	(.33, 1.00)		.05	0.57	(.30, 1.07)		.08

OR: odds ratio; CI: confidence interval.

*adjusting for age, (gender), BMI, education level, smoking status, smoking pack-years, alcohol drinking status, presence of hypertension, hyperlipidemia, and diabetes.
